# Visceral and Ectopic Abdominal Fat Effect on the Calcification of the Abdominal Aorta and Its Branches—An MSCT Study

**DOI:** 10.3390/life14010002

**Published:** 2023-12-19

**Authors:** Ivan Ordulj, Frano Šarić, Mirko Tandara, Kristian Jerković, Sanja Lovrić Kojundžić, Maja Marinović Guić, Miodrag Beneš, Danijela Budimir Mršić

**Affiliations:** 1 Clinical Department of Diagnostic and Interventional Radiology, University Hospital of Split, Spinčićeva 1, 21000 Split, Croatia; iordulj@gmail.com (I.O.); fsaric33@gmail.com (F.Š.); mirko.tandara@gmail.com (M.T.); jerkovickristian@gmail.com (K.J.); lovric.sanja@gmail.com (S.L.K.); maja.marinovic.guic@gmail.com (M.M.G.); 2 University of Split School of Medicine, Šoltanska 2, 21000 Split, Croatia; 3 University Department of Health Studies, University of Split, Ruđera Boškovića 35, 21000 Split, Croatia; 4 Institute of Public Health Sveti Rok Virovitica—Podravina County, 33000 Virovitica, Croatia

**Keywords:** visceral artery, iliac artery, visceral fat, aortic calcification, atherosclerosis, non-enhanced CT

## Abstract

Visceral and ectopic fat accumulation might have an impact on the atherosclerotic calcification of abdominal arteries. The pattern of calcification of the abdominal aorta and its branches is not fully investigated. We retrospectively analyzed the abdominopelvic MSCT images and calculated calcification volumes of the abdominal aorta, celiac trunk, superior and inferior mesenteric arteries, and both common and external iliac arteries. On the same MSCT scans, a visceral fat volume and ectopic fat deposits (liver-to-spleen ratio (L/S) and pancreas-to-spleen (P/S) ratio) were also measured. The results showed that calcifications of the abdominal aorta and its branches were associated with visceral fat volume, less strongly associated with L/S, and not associated with the P/S ratio. The abdominal aorta, the common iliac and external iliac arteries were more calcified arteries compared to the celiac trunk and superior and mesenterial arteries. In conclusion, visceral fat has a stronger effect on abdominopelvic arteries’ calcification than ectopic fat. Visceral aortic branches are generally less calcified than iliac arteries.

## 1. Introduction

Atherosclerosis denotes the accumulation of fatty and/or fibrous material in the intimal layer of arteries. Over time, the atherosclerotic plaques become more fibrous and accumulate calcium. Advanced atherosclerotic plaques can encroach upon the arterial lumen, impeding blood flow, which causes tissue ischemia. As a consequence, atherosclerotic cardiovascular disease (CVD) can develop in many organs and organic systems, leading to a high percentage of morbidity and mortality worldwide [1]. When it affects the heart’s own circulation, it can cause acute coronary syndromes, including myocardial infarction, or chronic conditions, such as stable angina pectoris. In brain circulation, it causes many ischemic strokes or transient cerebral ischemic attacks. Atherosclerosis can further lead to the formation of aneurysms, which commonly form in the abdominal aorta. When it affects the peripheral arteries, it can cause intermittent claudication, ulceration, and gangrene that can threaten the vitality of the extremities. Altogether, cardiovascular diseases, which include coronary heart disease, hypertension, and stroke, constitute the largest cause of death [2,3]. Understanding the pathophysiology of atherosclerosis is crucial in providing better prevention, diagnostic, and therapeutic strategies.

Among proven risk factors, obesity is very closely related to atherosclerosis. Numerous studies have demonstrated a relationship between obesity and atherosclerotic diseases. Both obesity and atherosclerosis are considered chronic inflammatory conditions, in which the activation of inflammatory processes by lipids plays a crucial role in triggering the disease. Distribution of fat is very important because visceral and abdominal fat are related to cardiometabolic risks of obesity [4]. Since 1980, the prevalence of obesity has doubled in more than 70 countries and continues to increase worldwide [3]. Several large cardiometabolic imaging studies, using computed tomography (CT) or magnetic resonance imaging (MRI), have shown that abdominal fat depots are heterogeneous and differentially associated with atherosclerosis and cardiometabolic risk [5,6]. The data from some epidemiological studies have shown that visceral adipose tissue was an independent marker of morbidity and mortality and that abdominal subcutaneous adipose tissue accumulation was a much weaker indicator of cardiovascular risk [7]. Emerging evidence also suggests that ectopic fat deposition, including hepatic, pancreatic, and epicardial fat, were linked with increased visceral fat deposition and therefore might also contribute to increased atherosclerosis and cardiometabolic risk [8].

As previously mentioned, arterial calcification is commonly seen in atherosclerosis, with coronary artery calcification being the most studied. Imaging methods can assess macrocalcification (assessed via the Agatston score) and microcalcification (assessed via the uptake of 18F-sodium fluoride in PET/CT scans) [9]. Although a high Agatston score predicts cardiovascular events, a heavily calcified plaque is usually stable plaque [10,11]. Arterial calcification was previously considered a passive degenerative process but is currently recognized to be a complex process actively regulated by various cell molecules [12]. The abnormal deposition of extra osseous calcium salt in the human body occurs most frequently in the arterial system and causes arterial calcification [13,14], mainly involving intima calcification and media calcification. However, these two types of arterial calcification cannot be accurately distinguished by conventional CT scans [15]. According to the literature, arterial calcification is associated with traditional cardiovascular risk factors such as diabetes, hypertension, hyperlipidemia, chronic kidney disease (CKD), and aging [16,17].

Several published studies investigated calcifications of the abdominal aorta and its branches with inconclusive results [18,19,20], and the clear effect of the visceral and ectopic fat on abdominopelvic arteries remained under investigated. We think that the non-contrast multislice CT (MSCT) scan of the abdomen and pelvis can reveal the associations of fat tissue accumulation and the atherosclerotic calcifications of abdominopelvic arteries. Therefore, on a cohort of polytrauma patients who underwent abdominopelvic MSCT examination, we examined the possible associations between three measures of abdominal fat and calcification volumes of eight abdominopelvic arteries.

## 2. Materials and Methods

### 2.1. Study Design and Participants

**Figure 1:**
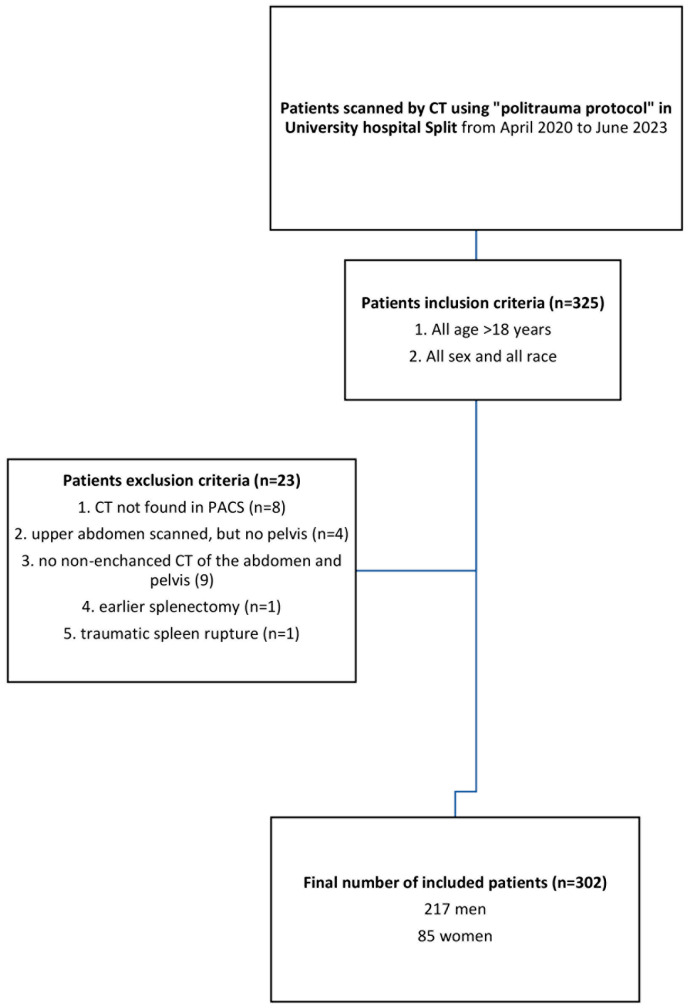
Flow chart of the study population included.

The retrospective cross-sectional study was conducted on randomly selected adult patients who underwent an urgent abdominopelvic CT scan requested from a Surgical Emergency Department of our hospital from 1 April 2020 to 30 June 2023. The MSCT scanning was performed using a 128-slice CT, Somatom, Siemens, Berlin, Germany. The data were retrospectively extracted from the Picture Archiving and Communication System (PACS). The age and sex of the patients were retrieved from the Hospital Information System (HIS). Patients with incomplete medical history or inadequate imaging documentation were excluded from analysis, as well as patients with lacking imaging data, as shown in detail in Figure 1.

### 2.2. Visceral Fat Measurement

**Figure 2:**
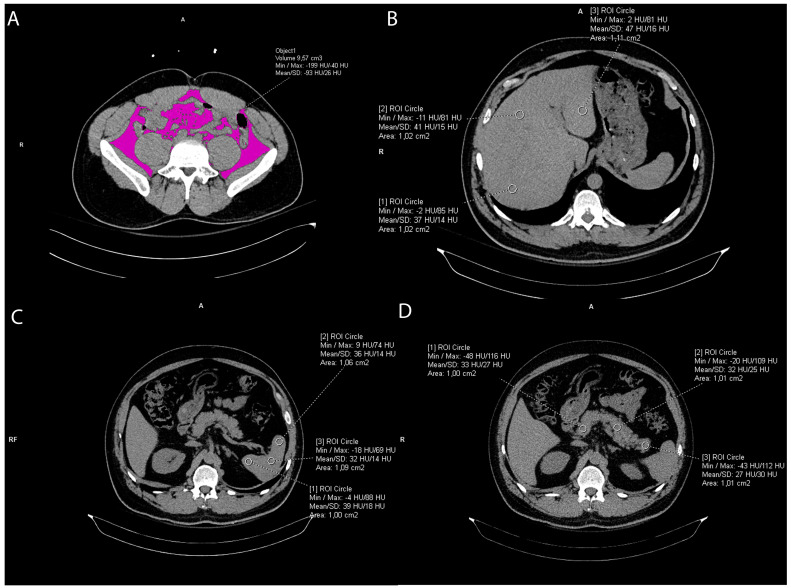
Representative axial CT images showing visceral fat volume calculation (**A**), liver attenuation measurement (**B**), spleen attenuation measurement (**C**), and pancreas attenuation measurement (**D**).

The intraabdominal visceral fat volume, expressed in cm^3^, was measured on the pre-contrast CT scan by manually drawing a line along the inner contour of the abdominal muscles which surround the abdominal cavity at the umbilical level. The CT value of fat tissue attenuation from −200 to −40 HU was semi-automatically segmented within the drawn line and the visceral fat volume was automatically calculated using the Siemens software *syngo*.via VB60A_HF08 (Figure 2).

### 2.3. Liver and Spleen Attenuation Measurement

**Figure 3:**
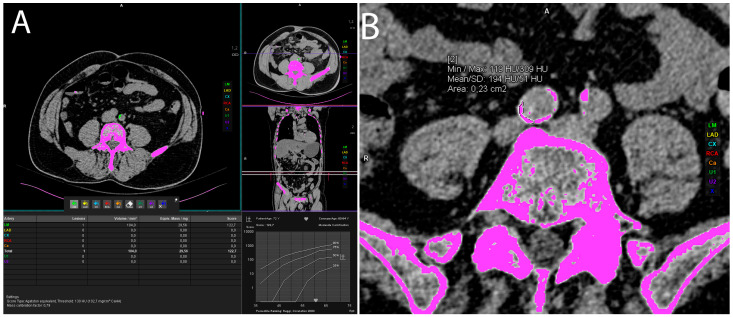
Representative axial CT images showing vascular calcification measurement by manually labeling every calcification in the artery of interest (**A**) or using freehand ROI method in case of larger calcifications (**B**).

Liver CT attenuation was estimated by drawing three round regions of interest (ROI) of the approximately same size, 1 cm^2^, at the portal vein level within the left lobe, right anterior lobe, and right posterior lobe of the liver, respectively. All ROIs were carefully distributed in the liver parenchyma and the biliary, vascular, and extrahepatic structures were excluded. (Figure 2). The final liver attenuation measure was an average Hounsfield Unit (HU) at the three measurements sites. Spleen attenuation was similarly obtained by averaging three manually placed round ROIs (approximately 1 cm^2^) at three different areas of the same slice (Figure 3). The attenuation index, the liver-to-spleen attenuation ratio (L/S), was calculated by dividing average liver with average spleen attenuation, fi L is the hepatic attenuation and S is the splenic attenuation [21,22]. The index was used as a measure of liver fat accumulation (ectopic fat).

### 2.4. Pancreas Attenuation Measurement

Five ROIs were drawn over the uncinate process, the head, neck, body, and tail of the pancreas (Figure 2), while three ROIs were drawn in the spleen, as previously mentioned (Figure 2), [23,24]. In both organs, a circular ROI of 1 cm^2^ was drawn [24,25]. To ensure reproducibility of the measurements, the ROIs were manually placed in order to avoid vessels and parenchymal calcifications. An average of the ROIs was calculated and then the pancreas-to-spleen attenuation ratio (P/S) was determined by dividing the mean attenuation value of the pancreas with the mean attenuation value of the spleen. The pancreas-to-spleen attenuation ratio significantly correlated with pancreatic fat accumulation (pancreatic steatosis, ectopic fat) proven histopathologically [24,25,26,27,28].

Intraclass correlation coefficients (ICC) showed excellent reliability of measurements: for the visceral fat volume, it was 0.982 (95% CI 0.96–0.99, *p* < 0.01); for the liver-to-spleen ratio, 0.939 (95% CI 0.80–0.97, *p* < 0.01); and for the pancreas-to-spleen ratio, 0.852 (95% CI 0.71–0.93, *p* < 0.01).

### 2.5. Vascular Calcification Measurement

Volumes of arterial calcifications were measured using Siemens *syngo*.via VB60A_HF08 software “Ca Scoring” technique, which estimates calcifications on unenhanced CTs, and it is routinely used for coronary artery calcification measurement and calculation of the Agatston score. Each researcher underwent training prior to the measurements. The procedure was as follows: an unenhanced abdominopelvic CT scan was opened in the “Ca scoring” evaluation *syngo*.via VB60A_HF08 software. First, the coronary calcifications were manually delabeled and then each vascular calcification was manually labeled in the artery of interest. There were eight observed arteries: abdominal aorta, celiac trunk—celiac artery (CA), superior and inferior mesenteric artery (SMA and IMA), left and right common iliac artery (CIA), left and right external iliac artery (EIA) (Figure 3). If the vascular calcifications were too close to the bone structures (spine) or were extending into the adjacent artery (for example, the abdominal aorta to the iliac artery), the freehand ROIs were used to delineate calcifications of interest and calculate the calcification volume (Figure 3).

### 2.6. Statistical Analysis

Statistical analysis was performed using programming language Python ver. 3.9, with Pingouin ver. 0.5.3 and SciPy ver. 1.11.3 statistical libraries used.

Correlations between two quantitative variables (fat measurements and arterial calcification volumes) were calculated using Spearman’s partial correlation. Due to the large number of partial correlations, the Bonferroni correction for multiple comparisons were used, which means that in partial correlations, the alpha value is equal to 0.00005 and *p* values lower than the alpha are considered significant. Partial correlation helped us check many connections between variables, taking into account confounding variables. If a borderline value for the Variance Inflation Factor (VIF) was found, it led us to choose which variable would be retained in the regression analysis. A multiple regression model was used to examine whether visceral fat, along with the L/S and P/S ratios, was a significant predictor of the volume of calcifications in individual arteries. Multiple regression was performed in statsmodels ver. 0.14.0. after testing for collinearity. Before each regression analysis, data standardization was performed within the scikit-learn library [29] and the displayed regression coefficients were all standardized. Variables of regression analyses were checked for multicollinearity using the VIF [30]. To compare medians between men and women for individual numerical columns, the Mann–Whitney U test was used, without Bonferroni correction (α = 0.05).

Finally, “observer biases” were minimized by having more than one observer. Observers were trained to take measurements at the beginning of the study and followed standardized procedures. The ICCs were obtained using the Pingouin library [31].

## 3. Results

The study population sample enclosed 302 participants, of which 85 (28.15%) were women and 217 (71.85%) were men. Patients were mostly of middle age (median age was 53), of which the women were somewhat older than the men (median 62 vs. 46 years, *p* < 0.001). There were no significant comorbidities in higher percent within the population group, of which the most common comorbidity was arterial hypertension. Ectopic fat (liver-to-spleen ratio and pancreas-to-spleen ratio) was not significantly different between women and men, but men had more visceral fat deposits (*p* < 0.0001). The most calcified observed vessel was the abdominal aorta with a mean volume of calcification of 1198.32 mm^3^ in a total population sample, and the calcification volume was significantly higher in women than in men (1533.15 vs. 1066.56 mm^3^, *p* < 0.0001). After the abdominal aorta, both common iliac arteries had the second highest calcification volume (right 377.54 and left 389.27 mm^3^), followed by both external iliac arteries (right 114.59 and left 83.13 mm^3^). In contrast to them, the anterior visceral branches, celiac trunk, AMS, and AMI had less calcification (22.56, 28.65 and 0.80 mm^3^, respectively) (Table 1).

**Figure 4:**
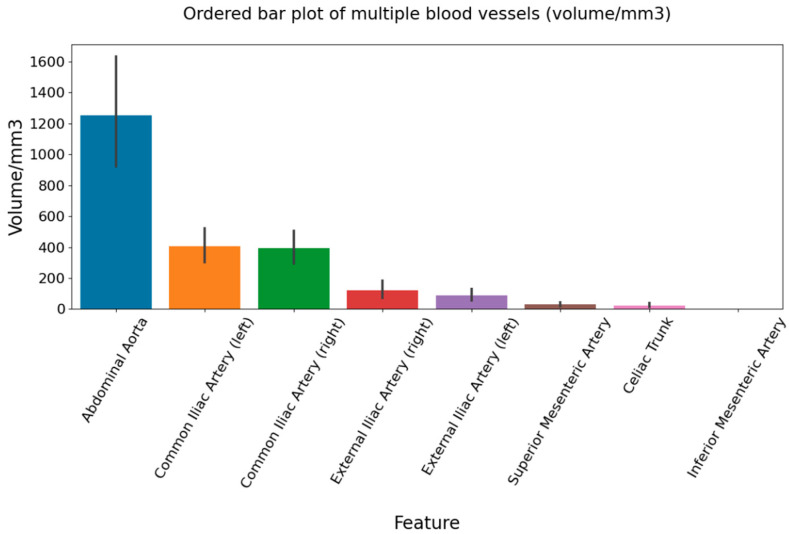
Bar graph shows calcification volume of observed abdominopelvic arteries.

Partial correlation among calcifications of abdominal aortic branches and abdominal fat measure were non-significant after taking confounding variables into account (not shown), in contrary to the significant correlations of the aorta and the majority of its branches, as well as of correlations among the fat measures, all presented in Table 2. The shown partial correlations were positive and moderate to strong. Only visceral and ectopic fat in the pancreas correlated negatively (Rho = −0.29). The calcification volumes of the abdominal aorta and its branches were shown as a bar graph (Figure 4). 

Multiple regression analyses with abdominal aorta, superior mesenteric artery, and common iliac artery calcification volumes as dependent variables and visceral and ectopic fat measures as independent variables, after removing the effect of other confounding variables, revealed that a significant independent predictor of abdominal aorta calcification was only visceral fat (*p* = 0.006, α = 0.05), in contrast to ectopic fat (*p* = 0.657, α = 0.05 and *p* = 0.903, α = 0.05 for the liver-to-spleen ratio and the pancreas-to-spleen ratio); R^2^ = 0.039, Table 3. Also, visceral fat was a significant independent predictor of common iliac artery calcification (*p* = 0.001), in contrast to the liver-to-spleen ratio and the pancreas-to-spleen ratio (*p* = 0.193 and *p* = 0.147), which were not; R^2^ for common iliac artery was 0.058. The visceral fat volume was a significant predictor of superior mesenteric artery calcifications (*p* = 0.013), and so was the liver-to-spleen ratio (*p* = 0.027), while the pancreas-to-spleen ratio was not (*p* = 0.143); with R^2^ of 0.047, Table 3.

Finally, a threshold for extensive aortic calcification was calculated according to the 90th percentile of the calcification volume of the abdominal aorta, Table 4. It increased by age and it was the highest in the ≥75 years age group (women 4989.11 mm^3^ and men 12,848.22 mm^3^). Also, the threshold for women was lower compared to men in almost all age groups.

## 4. Discussion

The results of the present study showed that calcification volumes of the abdominal aorta and its branches were associated with the visceral fat volume and that ectopic abdominal fat was less strongly associated. The abdominal aorta, followed by the common iliac and external iliac arteries, were more heavily calcified vessels, compared to the celiac trunk and superior and mesenterial arteries.

The abdominal aorta branches into several arteries in the abdomen, of which the present study investigated the anterior group of branches: the celiac trunk, superior mesenteric artery, and inferior mesenteric artery. These arteries supply the parenchymal abdominal organs (except kidneys and adrenals), small intestine, and large intestine. Both common iliac arteries branch/extend from the end of the aorta and they are further bifurcated into external and internal iliac arteries. Their role is to provide blood supply to the pelvic organs and lower extremities. Of the mentioned arteries, the study found the abdominal aorta to be the most heavily calcified vessel, which was also confirmed earlier. One study showed that 99% of calcification in the aorta was deposited in the intimal layer [32]. Calcification of the aorta contributes to the development of arterial stiffness, measured commonly via pulse wave velocity [33]. The amount of calcifications in the abdominal aorta increase with age and are proven to be associated with traditional cardiovascular risk factors, such as diabetes mellitus, body mass index (BMI), systolic blood pressure, and pulse pressure [34]. Obesity plays important role in the development of atherosclerosis. The traditional and most commonly used measure of obesity is a BMI; however, it cannot differentiate fat accumulation locations throughout the body, such as visceral, subcutaneous, or ectopic locations. Therefore, we thought that abdominal MSCT can precisely separate visceral fat deposits from fat deposits in the parenchymal organs of the abdominal region in order to explore and separate their possible effect on atherosclerotic calcifications of different arteries. Our results showed that visceral fat was positively associated with the abdominal aorta and its branches calcification and that ectopic liver deposits of fat (liver-to-spleen ratio) significantly contributed to the calcification volume of only the superior mesenteric artery, while pancreatic steatosis (pancreas-to-spleen ratio) did not have effect on any observed artery. This implies that pancreatic fat deposits, also called pancreatic steatosis, estimated via the pancreatic-to-spleen ratio, have no effect of calcification of the abdominopelvic arteries. So far, fat accumulation in the pancreas was shown to be associated with obesity and metabolic syndrome, but it was found to have roles also in other pancreatic conditions, such as in the development of diabetes, acute pancreatitis, or pancreatic cancer [35]. However, it seems that it has no particular role in the development of atherosclerosis, probably because pancreatic fat deposits are relatively small in comparison to the other ectopic fat deposits. We also observed a negative correlation between pancreatic and liver fat accumulation, which might be a reason for discordance in their effect. Non-alcoholic fatty liver disease is a common chronic liver disease associated with classical atherosclerosis risk factors such as obesity, dyslipidemia, diabetes, and metabolic syndrome, with systemic calcified atherosclerosis itself [36]. In the present study, liver fat accumulation showed significant association with a visceral aortic branch, the superior mesenteric artery, but not with the abdominal aorta. This might support the fact that ectopic fat deposits are not neglectable in the development of the atherosclerosis process, but certainly have a slighter effect than visceral fat deposits. The results might also suggest that the extent of calcification could depend on many factors, as well as it might possibly be site dependent.

Apart from the mentioned ectopic fat deposits, visceral fat was positively associated with vessel calcifications in the majority of studies [37,38,39]. Another study investigated other adiposity measures, BMI and body fat percentage, that were not associated with the SMA calcifications [40]. After analyzing the results of the mentioned studies and the interpretation of our results, we think that both the amount and location of fat tissue accumulation could be important in some steps of atherosclerosis development, but the exact role is not explained yet.

Further, we observed that seven other investigated arteries (three visceral and four pelvic branches) had different calcification volumes among themselves. In general, we noticed the calcifications’ volume decreased in the craniocaudal direction, of which the most calcified vessel was the abdominal aorta, then both common iliac arteries, followed by the external iliac arteries. The visceral branches of the abdominal aorta were generally less involved than the mentioned conduit (aorta and iliac arteries). Visceral branches had smaller calcification volumes; volume was the highest in the superior mesenteric artery, followed by the celiac trunk, and finally, the inferior mesenteric artery. The common iliac arteries are extensions of the abdominal aorta, which could be the reason for a relatively high amount of calcifications within their walls. Less than double of the calcifications were found in external iliac arteries compared to common iliac arteries. There is one study [19] which shows external iliac arteries to be less calcified than adjacent arteries (aorta, common iliac, and femoral arteries), which is similar to our result. The study of Lin et al. [40] showed patients with superior mesenteric artery calcification to have significantly higher levels of calcified atherosclerosis also in other arterial beds (celiac trunk, coronaries, thoracic aorta, abdominal aorta, and iliac arteries), with similar correlation coefficient values to our study. Although atherosclerosis is a generalized process, the predilection sites for atherosclerosis were found, such as coronary arteries, which might depend on hemodynamics and other factors [41]. The study of Jadidi et al. showed that the abdominal aorta and the iliofemoral arterial segment were the first to develop calcifications, which further spread proximally, and the ascending thoracic aorta was the last aortic segment to develop calcifications independent of vessel diameter [42]. We did not investigate the molecular mechanisms of calcifications; however, we think it is important to briefly mention them. Intimal calcification is associated with atherosclerotic plaques. It is a result of the interaction of lipids, macrophages, the proliferation of smooth muscle cells, pro-inflammatory cytokines, and molecules responsible for bone remodeling, which lead to the accumulation of calcium in the necrotic lipid components of the cells. Medial calcification is associated with aging, diabetes, hypertension, osteoporosis, kidney disease, and includes the process of differentiation of smooth muscle cells into osteoblast-like cells. Smooth muscle cells from different embryological origins may transdifferentiate differently into calcifying cells [43]. The molecular investigations can also explain the differences in the extent of calcifications among different vessels. Finally, we think our study attributed to the understanding of the less-commonly investigated arteries in the calcified atherosclerosis process and its association with obesity.

There are several limitations to address. First, the inability to measure all sites of ectopic fat accumulation, such as epicardial, perivascular, or intramuscular fat. Also, the study design was retrospective and cross sectional. However, there are also several strengths. Exhaustive measurement of not only the abdominal aorta calcification volumes, but also seven other abdominopelvic branches of the aorta that were not commonly investigated, will improve the understanding of calcification predilection locations and the extent of calcification within different arteries. The research included a relatively high number of patients. Fat accumulation was estimated using three abdominal sites of its deposition: two ectopic locations and one visceral location. 

## 5. Conclusions

To conclude, our study confirmed that the abdominal aorta was the most heavily calcified artery of the abdominopelvic region, followed by the common and external iliac arteries (the aortic extensions after bifurcation), while preceding visceral anterior aorta branches were less calcified. Also, we showed visceral fat to be an independent predictor of the mentioned arterial calcifications, in contrast to the smaller and probably non-significant effect of ectopic fat.

## Figures and Tables

**Table 1:** Baseline characteristics of population sample and median (Q1–Q3) values of observed parameters.

	Total	Women	Men	*p*
Age (years)	53 (32–64)	62 (49–72)	46 (20–59)	<0.0001
Sex (N, %)	302 (100%)	85 (28.15%)	217 (71.85%)	<0.0001
Smoking yes	61 (20.19%)	24	37	-
no	105 (34.76%)	27	78	
ex	26 (8.60%)	7	18	
NA	110 (36.42%)	27	84	
Arterial hypertension, yes	58 (19.20%)	26 (30.58%)	32 (14.74%)	-
Diabetes, yes	25 (8.27%)	13 (15.29%)	12 (5.52%)	-
Cholesterol, yes	38 (12.58%)	23 (27.06%)	15 (6.91%)	-
Liver-to-spleen ratio	1.30 (1.14–1.45)	1.28 (1.20–1.51)	1.30 (1.11–1.43)	0.099
Pancreas-to-spleen ratio	0.98 (0.80–1.05)	0.95 (0.82–1.02)	0.97 (0.80–1.06)	0.584
Visceral fat (cm^3^)	18.42 (10.70–27.74)	14.30 (8.11–19.88)	21.26 (12.85–30.02)	<0.0001
Abdominal aorta (vol./mm^3^)	1198.32 ± 2642.19	1533.15 ± 2560.95	1066.56 ± 2667.77	<0.0001
Celiac trunk (vol./mm^3^)	22.56 ± 97.20	34.02 ± 113.04	18.12 ± 90.14	0.050
Superior mesenteric artery (vol./mm^3^)	28.65 ± 110.88	29.12 ± 81.12	28.47 ± 120.72	0.660
Inferior mesenteric artery (vol./mm^3^)	0.80 ± 7.20	2.06 ± 12.75	0.30 ± 2.85	0.808
Common iliac artery, right (vol./mm^3^)	377.54 ± 866.64	481.35 ± 855.75	336.87 ± 869.44	0.038
Common iliac artery, left (vol./mm^3^)	389.27 ± 924.65	415.74 ± 761.92	379.03 ± 981.85	0.366
External iliac artery, right (vol./mm^3^)	114.59 ± 477.75	129.78 ± 557.07	108.64 ± 444.14	0.404
External iliac artery, left (vol./mm^3^)	83.13 ± 306.38	78.14 ± 304.26	85.09 ± 307.88	0.406

**Table 2:** Spearman correlation coefficients (Rho) among MSCT-obtained volume of calcifications (mm^3^) in observed arteries and measures of visceral and ectopic fat in a total population sample (α = 0.00005, Bonferroni adjustment for multiple comparisons).

Parameters		Rho	CI 95%	*p*
VF (cm^3^)	P/S	−0.29	[−0.4 −0.18]	<0.00005
L/S	P/S	0.37	[0.27 0.47]	<0.00005
CA (vol./mm^3^)	CIA, left (vol./mm^3^)	0.28	[0.17 0.39]	<0.00005
CA (vol./mm^3^)	EIA, left (vol./mm^3^)	0.29	[0.18 0.41]	<0.00005
SMA (vol./mm^3^)	EIA, right (vol./mm^3^)	0.32	[0.21 0.43]	<0.00005
AA (vol./mm^3^)	EIA, right (vol./mm^3^)	0.35	[0.24 0.45]	<0.00005
AA (vol./mm^3^)	SMA (vol./mm^3^)	0.36	[0.25 0.46]	<0.00005
AA (vol./mm^3^)	EIA, left (vol./mm^3^)	0.41	[0.31 0.51]	<0.00005
SMA (vol./mm^3^)	CIA, left (vol./mm^3^)	0.41	[0.31 0.51]	<0.00005
SMA (vol./mm^3^)	EIA, left (vol./mm^3^)	0.41	[0.31 0.51]	<0.00005
CA (vol./mm^3^)	SMA (vol./mm^3^)	0.42	[0.31 0.51]	<0.00005
CIA, left (vol./mm^3^)	EIA, right (vol./mm^3^)	0.42	[0.32 0.51]	<0.00005
CIA, right (vol./mm^3^)	EIA, left (vol./mm^3^)	0.47	[0.37 0.56]	<0.00005
AA (vol./mm^3^)	CIA, left (vol./mm^3^)	0.60	[0.52 0.67]	<0.00005
AA (vol./mm^3^)	CIA, right (vol./mm^3^)	0.61	[0.53 0.68]	<0.00005
EIA, left (vol./mm^3^)	EIA, right (vol./mm^3^)	0.68	[0.61 0.74]	<0.00005
CIA, left (vol./mm^3^)	CIA, right (vol./mm^3^)	0.78	[0.73 0.82]	<0.00005

Abbreviations: F—visceral fat; L/S—liver-to-spleen ratio; P/S—pancreas-to-spleen ratio; CA—celiac artery; SMA—superior mesenteric artery; AA—abdominal aorta; CIA—common iliac artery; EIA—external iliac artery.

**Table 3:** Multiple regression analysis of abdominal artery, superior mesenteric artery, and common iliac artery.

	Abdominal Artery		Superior Mesenteric Artery		Common Iliac Artery	
	Coefficient	*p* Value	Coefficient	*p* Value	Coefficient	*p* Value
VF	500.70	0.006	18.35	0.013	174.53	0.001
L/S	−78.9404	0.657	16.12	0.027	69.98	0.193
P/S	−22.99	0.903	−11.31	0.143	−82.92	0.147
	R^2^ = 0.039		R^2^ = 0.047		R^2^ = 0.058	

VIF was <5 for all variables, which implied there was no multicollinearity among predictor variables. Abbreviations: VF—visceral fat; L/S—liver-to-spleen ratio; P/S—pancreas-to-spleen ratio.

**Table 4:** Threshold (90th) of extensive calcifications in age and sex groups.

Age Groups (Years)	Sex	Calcification of Abdominal Aorta (mm^3^)
<45	men	0
	women	0.2
45–54	men	1691.87
	women	165.00
55–64	men	4001.60
	women	2384.47
65–74	men	8364.21
	women	4861.30
≥75	men	12,848.22
	women	4989.11

## Data Availability

Upon reasonable request.
